# Natural Protein Films from Textile Waste for Wound Healing and Wound Dressing Applications

**DOI:** 10.3390/jfb16010020

**Published:** 2025-01-10

**Authors:** Livia Ottaviano, Sara Buoso, Roberto Zamboni, Giovanna Sotgiu, Tamara Posati

**Affiliations:** 1 National Research Council, Institute for Organic Synthesis and Photoreactivity (CNR-ISOF), Via P. Gobetti 101, 40129 Bologna, Italy; livia.ottaviano@isof.cnr.it (L.O.); roberto.zamboni@isof.cnr.it (R.Z.); 2 Kerline srl, Via Gobetti 101, 40129 Bologna, Italy; sara.buoso@kerlinesrl.com

**Keywords:** silk fibroin films, keratin films, wound healing, wound dressing

## Abstract

In recent years, several studies have focused on the development of sustainable, biocompatible, and biodegradable films with potential applications in wound healing and wound dressing systems. Natural macromolecules, particularly proteins, have emerged as attractive alternatives to synthetic polymers due to their biocompatibility, biodegradability, low immunogenicity, and adaptability. Among these proteins, keratin, extracted from waste wool, and fibroin, derived from *Bombyx mori* cocoons, exhibit exceptional properties such as mechanical strength, cell adhesion capabilities, and suitability for various fabrication methods. These proteins can also be functionalized with antimicrobial, antioxidant, and anti-inflammatory compounds, making them highly versatile for biomedical applications. This review highlights the promising potential of keratin- and fibroin-based films as innovative platforms for wound healing, emphasizing their advantages and the prospects they offer in creating next-generation wound dressing devices.

## 1. Introduction

Wound management is a complex field that requires continuous effort in scientific research to develop more effective therapies, especially in cases where conventional treatments are insufficient. Wound dressings have evolved from traditional materials (e.g., plasters, gauze, cotton, and bandages), which primarily protect the wound surface from infection and contamination and require manual intervention to remove them, to more innovative and modern dressings that not only cover the wound but also accelerate the healing process. An ideal wound dressing should balance fluid absorption and release, effectively absorbing exudate while maintaining the necessary hydration for the healing process and preventing the dressing from adhering to the lesion. The materials used for wound dressings should be biocompatible, biodegradable, and provide thermal insulation and mechanical stability. Moreover, they should allow gaseous exchange between the injured tissue and the environment, facilitating oxygen penetration. This is critical for cell proliferation, angiogenesis, protein synthesis, and for inhibiting the growth of anaerobic bacteria [1,2,3].

A critical factor of wound healing is preventing the growth and colonization of harmful microorganisms, such as *E. coli*, *B. subtilis*, and *P. aeruginosa*, or fighting those already present. In healthy individuals, the immune system plays a key role in preventing infection by sending macrophages to the wound site, where they engulf pathogens through phagocytosis. However, if the immune system fails to eradicate these pathogens, infections can develop. Therefore, it is essential to create multifunctional wound dressings that support the body’s natural healing processes and address issues such as severe inflammation, scarring, and wound infection. Consequently, the structure of the requested dressing should provide an efficient platform for drug delivery, controlling the release of active molecules. A variety of materials, both synthetic and natural, are available for wound healing dressings (e.g., sponges, hydrogels, hydrocolloids, films, membranes), and the appropriate selection of materials significantly influences the effectiveness of the dressing. The material choice should be guided by a thorough understanding of wound dressing properties and an assessment of the material’s ability to adapt to the changing healing environment, taking into account factors such as wound depth, exudate volume, chronicity, and infection presence.

Tissue engineering primarily focuses on utilizing materials that can emulate and temporarily replace the native extracellular matrix (ECM), the most abundant component of the dermal layer. These materials must mimic the physicochemical properties of the ECM, providing inherent biological activity, cell adhesion, mechanical support, and a suitable structure for cell attachment, all without causing harm to the surrounding tissue [4]. Since the native extracellular matrix contains natural proteins and polysaccharides, biopolymers with similar characteristics—offering resistance and biological compatibility—have been proposed as promising biomaterials. These natural polymers can stimulate native tissue through specific interactions with cells, unlike synthetic polymers, which, despite their advantages in terms of reproducibility, are often biologically inert [5,6]. Hence, identifying biopolymers with wound healing capabilities and understanding their impact on fibroblast behavior represents a crucial opportunity to advance wound healing.

In this respect, recent research has focused on recovering protein fibers from textile industry waste, such as wool keratin (Ker) and silk fibroin (SF), as these materials offer valuable raw materials for developing innovative biomedical devices. These textile-derived biopolymers possess desirable properties, including strength, flexibility, biocompatibility, and biodegradability, making them ideal for supporting and promoting the wound healing process [7,8]. Reusing these materials has both economic and environmental benefits, as the technological processing of textile fibers generates large amounts of waste that can be reused, contributing to a greener and more sustainable society [9].

SF is a natural protein which can be extracted from the fibers of silkworms of *Bombyx mori* [10] and possesses several desirable features for biomedical use, including excellent structural properties, mechanical strength, controlled biodegradability, and non-inflammatory activity [11,12,13] The FDA has approved its use for sutures, tissue regeneration, coating devices, and drug delivery systems [14].

Ker is the principal structural protein of wool, nails, hair, horns, and feathers [15].

Its structure is rich in cysteine, which provides mechanical, thermal, and chemical stability, making keratin highly stable in water—a key advantage for biomedical applications. Additionally, its primary structure contains two motifs—“Arg-Gly-Asp” (RGD) and “Leu-Asp-Val” (LDV)—which facilitate cell adhesion by forming bonds with surface ligands on cells [16].

Interestingly, both of these polymers are characterized by high molecular weights (45–60 kDa for keratin extracted from wool and around 350 kDa for fibroin from *Bombyx mori* cocoons) that make these proteins particularly suitable for processing into several types of structural materials, such as sponges, electrospun fibers, nanoparticles, and films [17,18,19,20].

Among these various formats, films are particularly interesting as wound dressing materials due to the advantages they offer in terms of physical properties, including flexibility and adhesiveness, which enable easy conformation to hard-to-cover body areas [21]. More often these films are thin, transparent, and allow clinicians to monitor wound healing progress without removing the dressing. This minimizes trauma during dressing changes, reduces exposure to bacteria, and lowers the risk of infection.

This dressings category is frequently used in the medical sector for treating dry superficial wounds, minor burns, lacerations, and certain low-exudate ulcers, as they have a reduced capacity to absorb biological fluids due to their occlusive nature and their poor swelling properties. Hence, this limits their application for treating severe wounds with high exudates [2,22]. Films offer several advantages during different stages of wound healing. They provide a barrier to external contamination and to prevent blood loss and support the function of immune cells, cytokines, and growth factors. Moreover, films possess small pores that only allow the transmission of small molecules, such as oxygen, which are beneficial to prevent the invasion of microorganisms into the wound site and can be used for direct drug delivery at the wound site, consequently exerting a therapeutic effect [1]. In this regard, fabricating thin adhesive films loaded with active ingredients and nano systems could enhance their efficacy [23]. This review aims to provide an overview of the preparation and application of keratin- and fibroin-based films, which are among the most commonly used scaffolds in medical applications for wound healing.

## 2. Methods for Film Preparation

**Figure 1:**
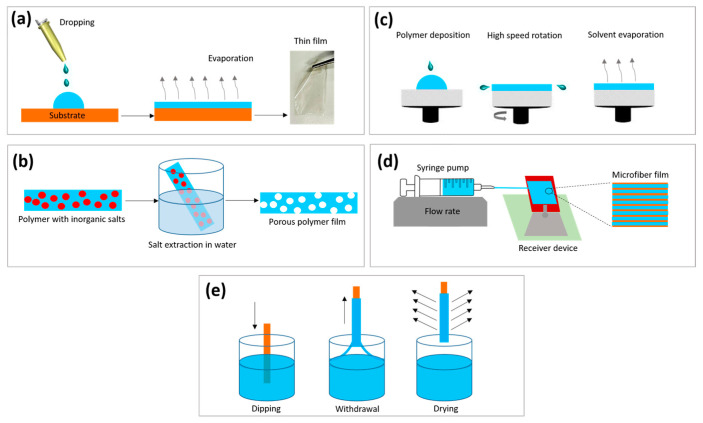
Schematic representation of the methods used for film preparation: (**a**) solvent casting; (**b**) salt leaching; (**c**) spin coating; (**d**) microfluidic spinning; (**e**) dip coating.

The preparation methods used for polymeric films have a direct impact on the properties of the final product. These techniques are customized to suit the specific polymers and their intended applications. The most commonly employed preparation methods include solvent casting, salt leaching, spin coating, microfluidic spinning, and dip coating (Figure 1). Each of these offers its own unique set of advantages and disadvantages in terms of film properties, scalability, cost-effectiveness, and suitability for different polymer systems and biomedical applications (Table 1).

### 2.1. Solvent Casting

Among the various film manufacturing techniques, solvent casting is preferred for its cost-effectiveness, simplicity, practicality, and ability to generate robust films with suitable mechanical properties and homogeneity [24].

This method involves the dissolution of biopolymers, in a suitable solvent and plasticizer (e.g., glycerin) if necessary, resulting in a suspension of different components with proper viscosity. The suspension is then cast onto a flat surface and dried without any external force except the temperature, allowing the solvent to evaporate and resulting in the formation of a polymeric film. After the drying process, the films are carefully removed and visually inspected for their integrity, color, and opacity and packed to maintain their stability (Figure 1a).

The rheological properties of the polymeric mixture must be carefully monitored during the casting and drying steps, as they significantly impact critical parameters such as the drying rate, morphology, and content uniformity of the resulting film [24]. This process has some limitations as the prepared films may become brittle upon storage, and the percent elongation may decrease due to the evaporation or loss of residual solvent within the film over time. Additionally, scaling up the production of films from a laboratory to a commercial level presents significant challenges. Achieving consistent film formation can be difficult, and variables such as heating, mixing speed, and environmental conditions may introduce inconsistencies in film quality across different production batches [24]. Furthermore, ensuring sterility throughout all the manufacturing steps is also a challenge [1].

### 2.2. Salt Leaching

The relatively smooth surface and low permeability of polymeric films can restrict their effectiveness in applications that would benefit from the increased permeability and improved nutrient transport provided by porous scaffolds; however, at the same time it is important to ensure that the porosity and pores do not compromise the mechanical properties of the films. To overcome this limitation, various methods have been used for the preparation of porous polymeric structures and porous films, such as for example the salt-leaching technique.

This technique is based on the insolubility of inorganic salts in organic solvents that can dissolve biodegradable polymers. The salts are added to the polymer solution before casting, serving as porogens. After casting, the polymer–salt composite is washed with deionized water to remove the salt crystals, and the resulting leached dressings are then dried to create porous wound dressings (Figure 1b). The porosity and pore sizes of the obtained porous structures can be varied by changing the salt-to-polymer ratios and the sizes of the leachable particles, respectively [25]. A large variety of inorganic salts, including potassium carbonate, sodium carbonate, sodium chloride, and ammonium bicarbonate, are commonly used as porogen agents [26].

However, this method has some limitations and cannot be applied to water-soluble materials for scaffold formation, as these materials may be removed alongside the leaching of the salt [27]. Additionally, the process of incorporating porogens can be problematic, due to the lengthy processing time and the possibility of residual porogens that could affect certain applications, particularly in medical and food-related contexts. In this context, an alternative was found in a study conducted by Jian Liu J. et al. [28]. In this study, micro/nano-sized pores were introduced into SF films through the use of SF nanoparticles generated by autoclaving and collected through ultracentrifugation. Specifically, methanol treatment was used to induce a β-sheet structure in the film while the embedded particles were extracted by water.

### 2.3. Spin Coating

Spin coating is currently the leading technique used to produce uniform thin films of organic materials, with thicknesses in the micrometer and nanometer range. The typical process involves depositing a small amount of an homogeneous polymeric dispersion onto a rotating substrate, which is accelerated to the desired speed, and the fluid is spread due to centrifugal force in uniform thin layers (Figure 1c). During the acceleration phase, the rotational motion causes a vigorous expulsion of fluid from the substrate, continuing until the fluid becomes thin enough to co-rotate with it [29].

Once the final speed is reached and the substrate spins at a constant rate, the fluid gradually thins out, initiating the drying phase. The rotation enhances evaporation, increasing the polymer concentration and the viscosity of the fluid, resulting in highly uniform films with a smooth surface.

The spinning speed and the viscosity of the dispersion can be adjusted to control the final thickness, allowing for a versatile process, even though it may not be highly efficient.

In the study conducted by Kamol Wasapinyokul et al. [30]. SF films with different numbers of layers were fabricated by the spin-coating method. All the samples exhibited high optical transmittance, regardless of the number of layers and length of heating time.

### 2.4. Microfluidic Spinning

Among these various approaches, microfluidic spinning is a promising technology that enables the precise manipulation of fluids within microscale channels. It is particularly suitable for volatile compounds that are sensitive to high voltages and temperatures, operating based on the principles of microscale fluid dynamics. This technique utilizes specially designed microfluidic devices with appropriate micro-channels. By injecting the solution at a specific flow rate, the core and sheath flows create a coaxial flow, allowing the polymer dispersion to be solidified into microfibers using methods such as UV light exposure, ionic or chemical crosslinking, and solvent exchange (Figure 1d) [31]. This method allows for the production of fibers in various shapes and sizes, which can be guided through a forward and reverse step process to create films. These films are then immobilized for a specific period at a controlled temperature to form the final product.

### 2.5. Dip Coating

As reported in the literature, the deposition of a uniform thin film of liquid over a substrate is most effectively achieved through the dynamic process of dip coating. This method involves three technical stages. Initially, the substrate is withdrawn vertically from the solution reservoir at a constant speed. During this withdrawal, a thin layer of the precursor solution is entrained on the substrate and begins to consolidate as it dries. Finally, the evaporation of the solvent—often accelerated by heated drying—leads to the formation of the deposited thin film [32]. In this context, SF has demonstrated its ability to function as a bioactive coating that can be absorbed onto various substrates, including established implant materials, while also enhancing their functionality [33]. The thickness and structure of the films are governed by the interplay of capillary forces (surface tension), viscous forces, and gravity. These factors can be modulated by altering the solution composition and the dehydration method. Furthermore, an optimized dipping protocol facilitates the deposition of uniformly assembled multilayer films on a substrate. This is achieved through the consecutive adsorption of the solution, interspersed with intermediate washing steps to remove unbound material [34].

However, it should be noted that the uniformity of the film’s properties is sensitive to turbulence during the deposition phase, which represents a significant limitation of this process.

The morphology and the physical characteristics of the resulting films are strongly influenced by the choice of solvent. SF and Ker films designed for biomedical applications have been produced using both aqueous and organic solvent systems [35], with the condition that the solvents are mild and biocompatible to ensure safety, avoid residual toxicity, and preserve the natural bioactive properties of these proteins.

For this reason, protein aqueous solutions are predominantly employed to create a film-castable solution [36,37,38]. Alternatively, various organic solvents can be used, with formic acid emerging as the preferred choice, particularly when blending SF and Ker with other polymers [39,40,41]. In fact, dissolving these proteins in formic acid results in transparent and remarkably stable solutions. According to the literature, formic acid can also influence the crystallization behavior of protein-based materials and increase the amount of beta-sheet structures [42,43]. This structural change can significantly impact on the enzymatic degradation rates of the material [44].

Moreover, to address the instability of dialyzed fibroin solutions, which tend to undergo gelation, certain applications require the use of hexafluoroisopropanol (HFIP). This solvent enables the preparation of solutions that remain stable over extended periods. However, due to the toxicity of HFIP, it is crucial to ensure complete solvent evaporation from the final films, guaranteeing that they are entirely free of HFIP residues before being used in topical applications. Additionally, it is essential to develop a closed-loop process to prevent the release of solvent vapors into the environment [45].

## 3. Fibroin and Keratin Films for Wound Healing and Wound Dressing Applications

SF and Ker solutions exhibit excellent film-forming abilities under all-aqueous processing conditions, and these have a high water and oxygen permeability and are particularly appealing as wound dressings and skin or corneal replacement grafts [46]. The water stability, biodegradability, mechanical resistance, and cell response of the films can be controlled by modifying the fabrication conditions [47], as well as adding plasticizer (such as dextrose and glycerol) and/or crosslinking agents [48].

The high demand for accelerated skin healing during the medical treatment of skin wounds is not fully met by the therapeutic effects of a single polymeric-based material. Incorporating additional functions, such as promoting cell growth and antimicrobial properties, into the scaffold would significantly simplify the clinical process and shorten the time required to achieve better therapeutic outcomes for skin wound healing.

Based on a literature search, we have compiled a list of research articles that have explored the clinical potential of SF and Ker films loaded with different active compounds to produce or increase a specific effect on the wound. These articles have been classified into groups based on their therapeutic effects.

### 3.1. Films with Antimicrobial Function

Bacterial infection is a major factor that hinders wound healing. While SF- and Ker-based dressings are beneficial for wound repair and healing, they lack inherent antibacterial properties to combat this issue. Additionally, these dressings may serve as a supportive base for bacterial growth. Therefore, given fibroin and keratin’s versatility and ability to be combined with other substances [49,50], incorporating antibacterial functions into biopolymer scaffolds is a promising strategy to improve their clinical use in treating open wounds and to decrease the risk of infections at the site. It is worth noting that, unlike SF, Ker already displays a mild antimicrobial effect, primarily due to the interaction between the positively charged amino groups in Ker and the negatively charged bacterial cell wall, which results in the inhibition of bacterial growth [51,52]. This is demonstrated, for example, in a study conducted by Sahil Goyal et al. [53]. The addition of Ker extracted from wool conferred antibacterial properties against *S. aureus* and *K. pneumoniae* to polymer films containing alginate and pectin, which showed no clear growth inhibition zone in the absence of the keratin.

Over the past decade, researchers have investigated various approaches to creating antimicrobial SF- and Ker-based biomaterials. In this context, a practical method is to develop infection-resistant biomaterials by integrating specific antibiotics into the protein matrix. This can be achieved by effectively blending antibiotics into aqueous or organic protein solutions before casting, resulting in antibiotic-loaded protein films. Aparna Yerra et al. [54] evaluated the addition of 11 different antibiotics to an SF solution, which was then used to produce thin films tested against specific pathogenic strains. The resulting antibiotic-based SF films demonstrated antimicrobial effectiveness, successfully carrying and delivering the drugs to the attached microbial cells, releasing them through passive diffusion. The localized drug delivery from biocomposite films offers a promising alternative to systemic antibiotic treatment, especially given the increasing concerns about microbial drug resistance that are important in clinical settings. In fact, this approach can provide a more targeted delivery, optimize pharmacokinetics, and reduce the required dosing frequency of drugs.

In response to the demand for effective alternatives, numerous studies have investigated the incorporation of various nanoparticles, microparticles, and natural compounds (including inorganic substances, honey, plant extracts, and natural polymers) as potential antimicrobial agents in wound dressings to improve their antimicrobial properties.

**Figure 2:**
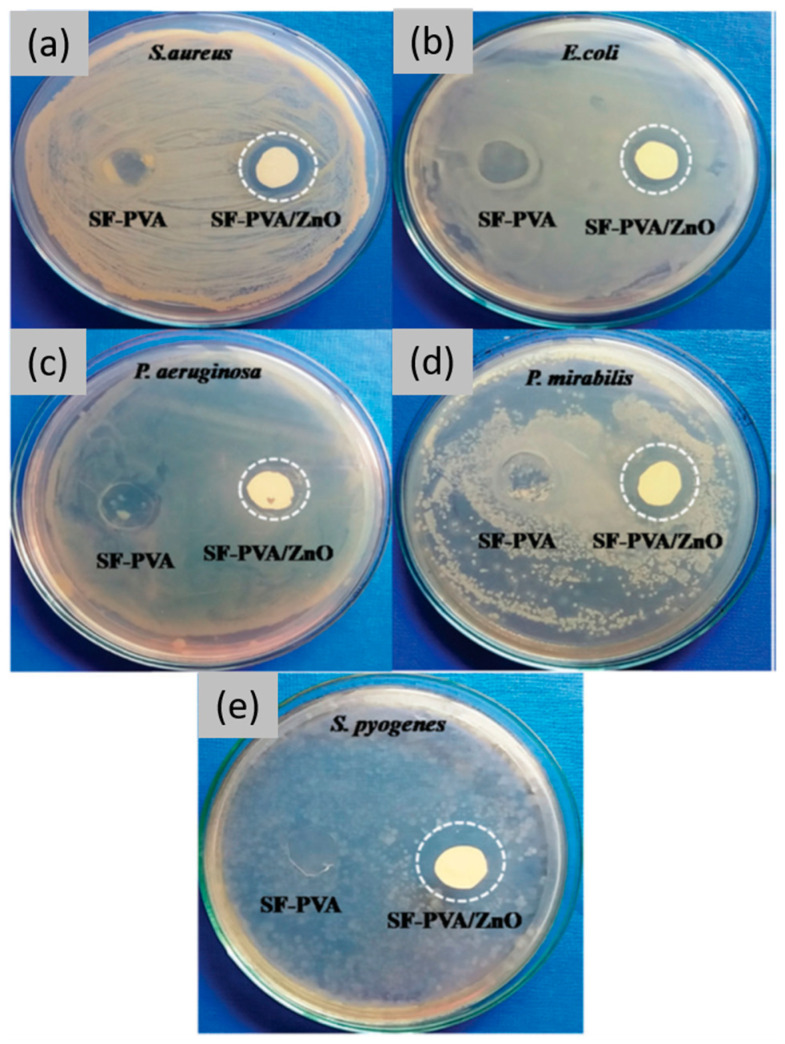
Antimicrobial activity of SF–PVA and SF–PVA/ZnO composite films against (**a**) *S. aureus*, (**b**) *E. coli*, (**c**) *P. aeruginosa*, (**d**) *P. mirabilis*, and (**e**) *S. pyogenes* microorganisms. Reprinted and adapted with permission from [57]. Copyright Royal Society of Chemistry 2018.

So far, the research has demonstrated that integrating small amounts of biocompatible nano-sized components, such as metal and metal oxide nanoparticles, within SF scaffolds can not only promote various cellular and molecular mechanisms that support the wound environment but also hinder bacterial adhesion and biofilm formation, thereby facilitating a more expeditious healing process [4,55]. Compared to antibiotics, the development of resistance in bacterial cells toward these nanomaterials is more challenging due to their multiple mechanisms of action. Inorganic nanomaterials primarily eliminate invading bacteria by generating reactive oxygen species (ROS), since high ROS levels can overwhelm the bacteria’s antioxidant defence mechanisms, causing oxidative damage to essential cellular components such as enzymes, proteins, DNA, and lipids. Besides ROS generation, inorganic nanomaterials employ additional bactericidal mechanisms, including disrupting the cell wall, damaging chromosomes and DNA, and interfering with the metabolic activities of microorganisms [55,56]. Priyanka P. Patil et al. [57,58] developed nanocomposite films made of SF–polyvinyl alcohol embedded with ZnO NPs for use as a dressing material. In vitro evaluation showed that these films exhibited antimicrobial activity against both model Gram-positive and Gram-negative bacteria. In addition, the zone of inhibition (ZOI) increased with higher ZnO nanoparticle content in the composite films (Figure 2). Silver nanoparticles (AgNPs) are also a type of nanoparticle that has attracted considerable attention due to their excellent electrical conductivity, remarkable chemical stability, and powerful catalytic and antibacterial properties [59,60,61]. Smita Patil et al. [62] reported the development of antibacterial SF films containing in situ synthesized AgNPs with antimicrobial properties and activity against both sessile and planktonic *S. aureus*, as well as against the biofilm formation of antibiotic-resistant *E. coli*. In addition, AuNPs functionalized with 4,6-diamino-2-pyrimidinethiol (DAPT) were combined with fibroin to prepare films that demonstrated excellent antibacterial capabilities for wound healing applications [63].

Particularly relevant is the use of a natural compound such as honey as an antimicrobial agent. Honey is a concentrated, viscous solution of floral sugars, proteins, enzymes, and amino acids derived from bee crops. Nowadays, there is a more comprehensive scientific understanding of how honey’s beneficial properties facilitate wound healing and regeneration [64,65,66,67]. Specifically, its well-documented antibacterial property can vary across different types of honeys, and it is influenced by various factors working either singularly or synergistically [68]. In most honeys, it can be attributed to the action of the endogenous enzyme glucose oxidase from the bee’s crop. This enzyme slowly breaks down glucose into gluconic acid, lowering the pH of honey and producing hydrogen peroxide, which sterilizes the wound and stimulates vascular endothelial growth factor production [69,70,71]. In the study conducted by Monika Rajput and colleagues [72], self-standing patterned and flat SF membranes containing honey were fabricated. It was observed that honey altered the physical properties of the membranes; specifically, its hydrophilic nature enhanced their wettability and water absorption capacity, resulting in increased swelling. This high degree of swelling indicates the membranes’ potential to absorb nutrients from the surrounding media, which can nourish the cells and promote favourable cell adhesion and proliferation. Indeed, a biocompatibility assay suggested that the incorporation of honey into SF membranes increased the adherence, proliferation, and viability of cells at higher honey concentrations and also enhanced cell–cell and cell–matrix interaction. Furthermore, honey serves as a nutritional factor, as it is gradually released from the membranes into the surrounding environment, providing nourishment to the cells. Another research study conducted by N. Sukumar et al. [73] focused on combining SF with honey and with epidermal growth factor (rhEGF) in order to promote the process of healing of diabetic wounds. The authors demonstrated once more that honey exhibits an extraordinary ability to promote the wound healing process and great antimicrobial activity against *E. coli* and *Staphylococcus epidermidis* (*S. epidermidis*).

An alternative approach to achieving films with antibacterial properties is combining different antimicrobial natural polymers [74]. For example, in the work of P. Ganesan et al. [8], honey was combined with SF, wool Ker, and chitosan (CS) to produce, using a casting technique, curative films capable of acting as drug-releasing agents on the wound surface and protecting the wound from secondary bacterial infection. Indeed, all the different dressings demonstrated good antimicrobial activity against both Gram-positive and Gram-negative bacteria such as *Staphylococcus aureus* and *Escherichia coli*. In the work of Meghann Rosewald et al. [75], chitosan was used alongside cellulose to produce Ker composites. These materials have demonstrated the ability to maintain the antibacterial and anti-inflammatory properties provided by the Ker and CS components. Furthermore, the composites have exhibited enhanced mechanical properties, including a significant increase in tensile strength, which allows them to be utilized in a broader range of practical and general applications. Additionally, Chieu D. Tran et al. [76] evaluated whether composites made of Ker, cellulose, and/or CS could encapsulate and control the release of a broad-spectrum antibiotic such as ciprofloxacin (CPX). All three biopolymers were capable of encapsulating the drug and subsequently releasing it at different rates, depending on the composition of the single-, two-, or three-component systems. In particular, it was found that the drug release rate can be controlled and adjusted at any desired rate by carefully selecting the appropriate concentration of Ker in the composite materials. This is due to keratin’s structurally denser nature, in contrast with the more porous structure of cellulose and chitosan, which leads to a much faster drug release rate from those materials. By combining all three components into a composite, it is possible to integrate their respective properties and create a high-performance dressing that can effectively heal wounds, kill bacteria, and deliver drugs for the treatment of chronic ulcerous wounds in diabetic patients.

Polymeric scaffolds can also be functionalized by incorporating various phenolic compounds derived from diverse plant extracts, which have been reported to be used in the development of bactericidal protein-based biomaterials [77].

**Figure 3:**
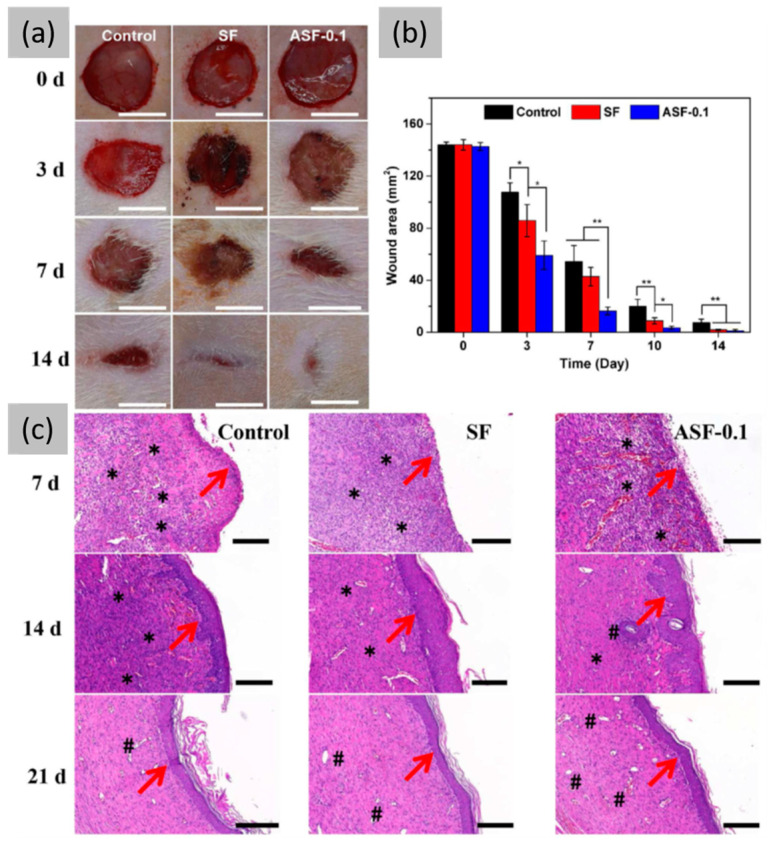
Wound healing ability of different films in a rat full-thickness wound model. (**a**) Representative photographs of the wound healing process over 2 weeks (scale: 10 mm). (**b**) Quantification of wound areas at days 0, 3, 7, 10, and 14 after operation. Note: * *p* < 0.05, ** *p* < 0.01. (**c**) Representative HE (hematoxylin and eosin)-stained photographs of the wound tissue regeneration process at 7, 14, and 21 days after surgery (scale: 200 µm). Note: →, new epithelium; *, inflammatory cells; #, hair follicle cells. Reprinted and adapted with permission from [79]. Copyright Elsevier Clearance Center 2023.

Additionally, antimicrobial peptides (AMPs), which are produced by bacteria, insects, plants, invertebrates, and vertebrates, are gaining attention as alternatives to traditional antibiotics [78]. To decrease their cytotoxicity and improve their antimicrobial stability, AMPs are often immobilized on the surfaces of specific materials. SF and Ker are considered some of the most promising candidates for surface functionalization due to their rich variety of active groups, such as carboxyl, hydroxyl, and amine. For example R. Si et al. [79] developed a novel wound dressing by immobilizing the marine-derived antimicrobial peptide actinomycin X2 onto SF fibers and subsequently developing a versatile SF film (ASF) with intrinsic antibacterial and angiogenic properties. In vivo experiments on bacterially infected wound healing showed that the antimicrobial films (AMFs) prevented wound inflammation, facilitated repair, and enhanced the wound microenvironment, demonstrating that ASF film could be a promising candidate for skin wound healing (Figure 3).

**Figure 4:**
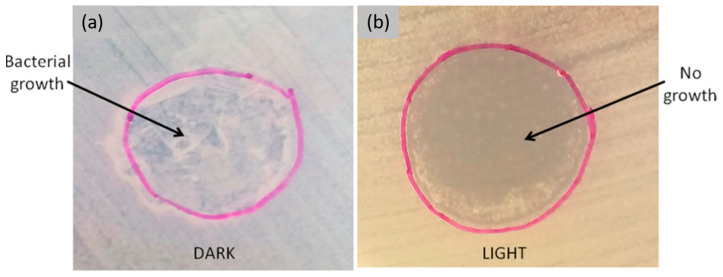
Antimicrobial “patch assay”. KFMB400 film placed upon LB agar inoculated with *S. aureus* was incubated in the dark (**a**) or irradiated (**b**) for 75 min. After film removal, bacteria were grown for 24 h at 37 °C. Reprinted and adapted with permission from [80]. Copyright Clearance Center American Chemical Society 2015.

Another promising alternative to conventional antibiotic-based therapy is antimicrobial photodynamic therapy (APDT). This approach combines the use of visible light, photosensitizers, and oxygen to generate singlet oxygen and other reactive oxygen species, which can effectively kill bacteria. Aluigi et al. [80] selected methylene blue, a highly effective antimicrobial photosensitizer, to dope Ker films and create new biodegradable, biocompatible materials for tissue engineering and wound healing. It was demonstrated that these materials can exert antimicrobial photodynamic activity when exposed to visible light (Figure 4). A summary of the different antimicrobial agents combined with SF and Ker has been reported in Table 2.

### 3.2. Films with Antioxidant Function

Over the past few decades, by combining knowledge from biomaterials and skin tissue regeneration, researchers have developed intelligent wound dressings with antioxidant properties that regulate ROS and facilitate skin tissue recovery. These biomaterials have emerged as innovative solutions for medical applications, particularly in treating skin injuries. While physiological levels of ROS are beneficial for timely wound healing, excessive production can negatively impact the healing process. Under non-physiological conditions, the persistent overproduction of ROS, which is not adequately balanced by the body’s antioxidant defenses, results in uncontrolled oxidative stress and has been linked to impaired healing in chronic, non-healing wounds [81,82].

Antioxidant compounds can help prevent or inhibit oxidation by stabilizing, deactivating, or scavenging free radicals that can damage cells. Therefore, integrating antioxidants into polymeric biomaterials, which typically have limited radical-scavenging abilities, offers a promising strategy to inhibit molecular oxidation and restore normal physiological levels of reactive oxygen species. Furthermore, this approach aims to improve the efficiency of these antioxidants, which often suffer from low bioavailability and bioactivity when directly administered onto the wound [83]. These compounds are present in various plants and may accelerate wound healing, as demonstrated in a study conducted by Tingting Luo et al. [84]. In this study, three natural antioxidants, vitamin C (VC), epigallocatechin gallate (EGCG), and curcumin, were physically incorporated into three types of silk films. The strong interaction between SF and the antioxidants not only preserved the structural integrity of the films but also stabilized the antioxidants, maintaining their activity for up to 14 days at 37 and 45 °C, compared to controls with free antioxidants in solution.

**Figure 5:**
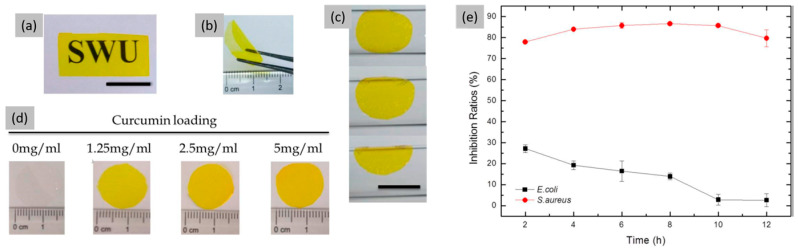
(**a**) Transparency of curcumin-loaded SF film (scale bar 10 mm), (**b**) free-standing curcumin-loaded SF film in the dry state, (**c**) hydrated films follow surface contours (scale bar: 10 mm), (**d**) SF films can be loaded with a range of curcumin concentrations, (**e**) inhibition ratio kinetic curves of SF/Gly/GA/Cur composite film against *S. aureus* and *E. coli*. Reprinted and adapted with permission from [46].

In this scenario, numerous studies have investigated the feasibility of developing various topical formulations loaded with curcumin, to overcome the poor bioavailability observed when this compound is directly applied to wound sites [85]. The wound healing potential of this natural polyphenolic molecule is primarily attributed to its antioxidant and anti-inflammatory properties. This is due to its ability to position itself within the cell membrane and act as a potent scavenger of various reactive oxygen species, including hydroxyl radicals and nitrogen dioxide radicals [86,87]. Moreover, curcumin exhibits strong antimicrobial properties [88] both on its own and in synergy with antibiotics, thereby protecting wound tissue from bacterial infections. Curcumin also promotes cell proliferation, aiding in the repair and reconstruction of damaged tissue [89]. Thus, given the proven ability of this compound to effectively accelerate the wound healing process by targeting various stages of the natural wound healing cascade, developing a curcumin-based wound dressing represents a promising strategy to optimize the targeted delivery and therapeutic benefits of this compound for skin wounds [85,90]. In this contest, Zhang and co-workers [46] prepared sustained-release film by blending curcumin with SF using the solution casting method (Figure 5). The results of this study indicate that the curcumin-loaded SF film is highly suitable for wound healing applications. It not only creates a moist environment for wound areas but also demonstrates effective sustained-release performance and impressive antibacterial activity against *S. aureus*, thereby protecting the wound from microbial invasion. Furthermore, in the study of Chunmei Li et al. [91], it is reported that curcumin, when physically bound to SF films, significantly enhanced the proliferation and differentiation of human mesenchymal stem cells when compared to free curcumin (in monomer or aggregate form) in cell culture medium.

Recently, our research [92] has demonstrated that bioactive and eco-sustainable films made by combining SF with antioxidant compounds extracted from pomegranate waste have a high capacity to reduce oxidative stress in cells. These transparent and flexible films have shown biocompatibility with the main skin cells (keratinocytes and fibroblasts) and were able to release bioactive compounds in a controlled manner, based on Fickian diffusion.

An important cause of ROS generation is the photochemical interaction of UVA radiation with biological materials, specifically with intracellular chromophores. To address this issue, Binbing Chen et al. [93] demonstrated that incorporating wool Ker into silk sericin films led to a microstructural transformation and a reinforcement of the molecular network, which conferred to the films an improved UV absorption and excellent anti-UV light properties. It must also be considered that exposure to oxidizing agents like UVA or certain pathological conditions can severely disrupt intracellular iron homeostasis, which plays a key role in both oxidative stress and photo-induced skin damage [94,95]. In this regard, Anastasia Anceschi et al. [96] developed novel bioactive films capable of binding iron in chronic wounds. These films were created by casting an aqueous solution of wool-derived keratosis (the soluble fraction of keratin), and their stability and water insolubility were increased through a thermal crosslink treatment. The utility of these dressings stems not only from their iron-binding ability but also from their capacity to swell and remove exudate from the wounds. A summary of the different antioxidant agents combined with SF and Ker has been reported in Table 3.

### 3.3. Films with Growth Factors

Growth factors are essential signaling molecules that play a key role in wound healing by enhancing wound closure, promoting the formation of granulation tissue, and facilitating the development of new blood vessels. Although the body naturally produces various growth factors, certain pathological conditions, like diabetes, which is marked by an impaired microcirculation, hinder the effective delivery of these bioactive compounds needed for chronic wound healing. This results in the development of chronic non-healing wounds where re-epithelialization fails to occur. Consequently, it is generally believed that providing a continuous supply of growth factors to the localized wound area through a slow-release drug delivery system would be advantageous for chronic wound therapy and for the clinical applications of tissue engineering [97]. However, the delivery of these compounds is challenging, as many exhibit a relatively short half-life in vivo, presumably due to enzymatic degradation within the wound bed. For this reason, to maintain an effective concentration of these growth factors, a formulation combining them with a suitable protein-based drug delivery system is recommended. In particular, SF has demonstrated excellent biocompatibility as a delivery platform, and the functionalization of different SF forms with growth factors provides a synergistic approach to improve wound healing. This combination takes advantage of both the structural support provided by the SF matrices and their capability to extend the half-life of the growth factors complexed with them, as well as their biological activity. This approach can accelerate the healing process by promoting tissue regeneration and reducing healing times.

**Figure 6:**
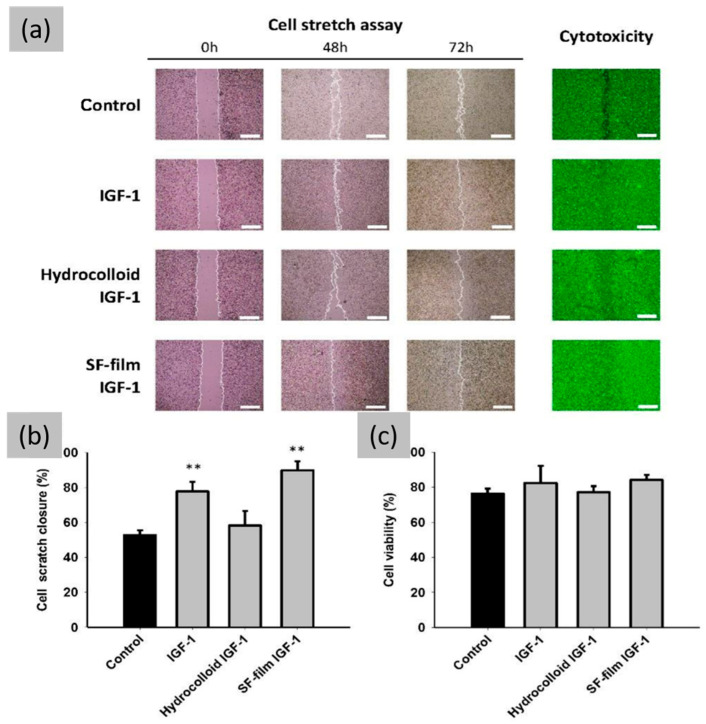
Effects of IGF-1, hydrocolloid IGF-1, and SF film IGF-1 on BALB/3T3 fibroblast scratch closure, cytotoxicity, and viability. (**a**) Scratch closure and cytotoxicity of BALB/3T3 monolayer in the presence of hyperglycemic medium at different times. Cells were stained with LIVE/DEAD stain and examined under a fluorescence microscope. (**b**) Quantification of cell scratch closure after different treatments at 48 h in hyperglycemic medium. (**c**) Cell viability after different treatments at 72 h. Significant differences between the control (black bar) and treatment groups were determined by Dunnett’s multiple comparison post hoc test. ** *p* < 0.01; n = 3; mean ± SEM. (Scale bars = 500 μm). Reprinted and adapted with permission from [99].

A study conducted by Eun Seok Gil et al. reports on the incorporation of epidermal growth factor and silver sulfadiazine, a widely used topical antimicrobial agent for preventing and treating wound infections, into silk biomaterials to assess their impact on wound healing [98]. Three types of silk biomaterials (porous films, electrospun mats, and non-porous silk films) were developed using two different drug functionalization techniques to assess the influence of silk material architecture and drug functionalization on wound healing. The study showed that silk biomaterials, likely due to their natural biocompatibility, ability to incorporate growth factors, controlled release properties, and potential to form porous structures, were able to improve the wound healing process. Additionally, Meng-Jin Lin et al. investigated the potential benefits of locally administering and sustaining the release of insulin-like growth factor-1 (IGF-1) from an SF film for patients with chronic, hard-to-heal wounds [99]. In fact, IGF-1 is a well-known stimulator of keratinocyte growth and migration, which are crucial characteristics for the re-epithelialization of wounds [100]. SF films demonstrated an excellent ability to deliver IGF-1; moreover, the cell growth-promoting activity of IGF-1 was maintained even after it was complexed with the SF films, which also significantly prolonged its half-life (Figure 6) [101].

In addition to direct incorporation, research has recently moved toward other methods that provide a significant supply of silk material containing growth factors, using genetic engineering technology, as investigated in the study of Sheng-Lan Wang et al. [102]. Specifically, advancements in transgenic technology have enabled silkworms to express human growth factors (FGF, TGF-β1, PDGF, VEGF, and EGF) within their silk glands, allowing them to synthesize genetically engineered cocoons that can be processed into a range of functionalized silk-based biomaterials.

On the other hand, the literature data suggest that keratin-based products have a stimulatory beneficial effect on their own, accelerating wound healing, particularly in terms of re-epithelialization. This is achieved by enhancing keratinocyte migration and the production of basement membrane proteins, such as types IV and VII collagens [103]. A summary of the different growth factors combined with SF and Ker has been reported in Table 4.

### 3.4. Films with Anti-Inflammatory Agents

While inflammatory responses by immune cells are essential for protecting against infections, excessive inflammation can be harmful. Macrophages, in particular, play a critical role in tissue injury and repair, significantly affecting the dynamics of wound healing. Their polarization into either an inflammatory (M1) or anti-inflammatory (M2) phenotype is crucial for pathogen clearance and promoting wound repair. However, the chronic activation of either macrophage type has been linked to various diseases, highlighting the need for localized treatments to address chronic macrophage activation.

**Figure 7:**
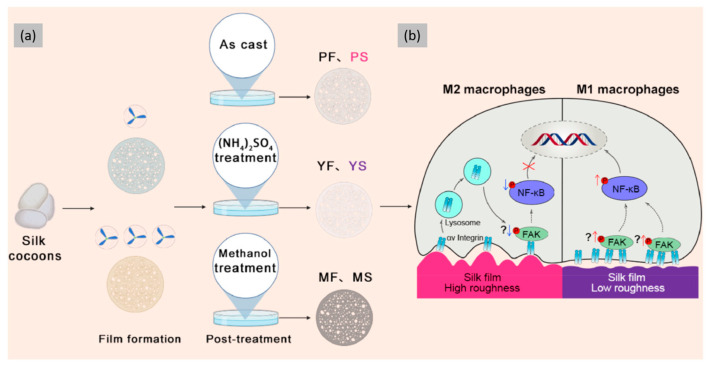
(**a**) Preparation and screening of SF films with different surface roughnesses and (**b**) the potential mechanism of roughness on macrophage polarization. Reprinted and adapted with permission from [104]. Copyright Elsevier Clearance Center 2024.

In this context, the design of wound dressings is particularly important. For example Doudou Hu et al. demonstrated that the physical properties of medical materials, especially their surface topography, play a crucial role in modulating intracellular signaling in macrophages [104]. In fact, SF films with a varying and tunable surface roughness, produced by slow-drying casting and salting out techniques, were able to induce differential macrophage polarization (Figure 7). This established a direct connection between the specific surface morphology of the SF films and the macrophage response, subsequently influencing tissue regeneration. Mechanically, the rough surface of the SF film enabled a positively curved membrane of macrophages, promoting the internalization and degradation of integrin αV and thus inhibiting the integrin–NF-κB signaling pathway, while SF film with a low roughness activates the integrin–NF-κB signaling pathway. Another approach to control the polarization of macrophages and the biological response, optimizing regenerative needs while reducing inflammatory pathways, was shown in the study of Andrew R.D. Reeves et al. [105] The authors used silk protein to fabricate biopolymer films that released either IFN-γ or IL-4 to modulate macrophage polarization. By varying the duration of the films’ exposure to water vapor, they were able to tune the solubility of the SF films and regulate their β-sheet content, enabling a short-term release of the respective cytokines. The released IFN-γ or IL-4 induced the polarization of THP-1-derived macrophages into the M1 or M2 phenotypes, respectively.

Furthermore, to modulate the inflammatory process, SF films can be designed as a delivery system for neurotensin (NT), a neuropeptide that acts as an inflammatory modulator in wound healing but is easily metabolized in biological environments [106]. Incorporating neurotensin into an SF film could be beneficial, as the film can protect the neuropeptide from degradation and sustain its release, prolonging its therapeutic effects. Notably, NT has been shown to effectively reduce the inflammatory status of wounds and promote fibroblast migration to the wound site, ultimately facilitating the expression of extracellular matrix (ECM) proteins essential for skin repair [107].The study by Camila Nunes Lemos et al. [108]. developed SF films that successfully protected the peptide from rapid degradation, allowing it to have the desired effect. Additionally, the application of iontophoresis in the reported films promoted a rapid release of NT, ensuring its immediate availability for action and providing a high concentration of the drug in the wound environment at the beginning of the treatment.

**Figure 8:**
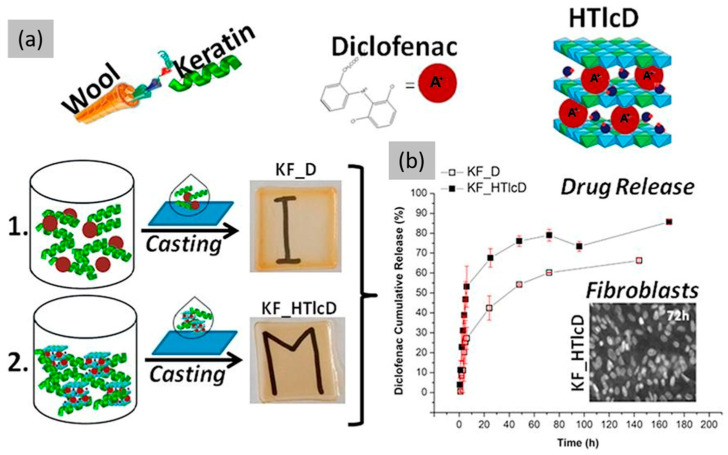
(**a**) Preparation of Ker films loaded with HTlc and diclofenac (KF_D and KF_HTlcD, respectively) and (**b**) comparison of in vitro release profiles of KF_D and KF_HTlcD. Reprinted and adapted with permission from [109]. Copyright Elsevier Clearance Center 2018.

Another approach to address this issue is to formulate drug-loaded films capable of managing inflammation and pain. For instance, our research [109] described the development of transparent and free-standing organic–inorganic hybrid films for drug delivery purposes. Specifically, the researchers created a matrix of Ker extracted from wool filled with ZnAl hydrotalcite (HTlc) nanoparticles that had intercalated diclofenac, a member of the non-steroidal anti-inflammatory (NSAID) group of drugs (Figure 8). Previous studies have also shown that diclofenac-incorporated formulations show antimicrobial activity, to some extent, against various bacterial strains [110,111,112]. The composite films were characterized and evaluated in vitro as drug delivery systems, since the incorporation of the anionic drug into the lamellar structure of the hydrotalcites enabled its protection and controlled release from the polymer matrix.

The work of Li Cui et al. [113] also involved the preparation of Ker films loaded with diclofenac. In order to maintain the mechanical strength and to improve the chemical stability of the materials, Ker was crosslinked with transglutaminase (TGase). In fact, this treatment led to the formation of new covalent bonds between the keratin proteins, resulting in the increased tensile strength of the films, along with a decreased elongation at break and reduced solubility in various solvents. Furthermore, the enzyme treatment resulted in a lower drug release rate from the films. A summary of the different anti-inflammatory agents combined with SF and Ker has been reported in Table 5.

### 3.5. Advantages and Disadvantages of Keratin- and Fibroin-Based Films as Wound Dressings

The use of natural proteins like Ker and SF for wound dressing applications offers numerous benefits, but also presents some limitations that require attention for practical implementation. Below, we have summarized and highlighted these aspects.

*Advantages.* Keratin and fibroin are derived from renewable resources such as wool and silkworm cocoons, making them environmentally friendly materials. Their biocompatibility ensures minimal immune responses, while their biodegradability facilitates gradual degradation in accordance with the wound healing process, without the need to remove the dressing. This reduces trauma to the wound and minimizes the risk of infection [1,2,21,46]. A key advantage of these proteins lies in their ability to support cell adhesion [16]. Ker contains specific sequences, such as RGD and LDV motifs, that promote cellular interactions, while SF’s hydrophilic nature enhances its compatibility with biological tissues. This makes both materials excellent candidates for creating a supportive environment for cell growth and tissue regeneration. Moreover, Ker and SF exhibit remarkable versatility in processing, allowing their transformation into films, nanofibers, and sponges [17,19]. This adaptability enables customization for various wound scenarios, whether it involves creating flexible materials for joints or thicker layers for wounds with high exudate. Lastly, both proteins can also be functionalized with several active compounds such as the above-mentioned antimicrobial, antioxidant, or anti-inflammatory agents [18,53].

*Disadvantages.* Despite their many advantages, Ker and SF also present challenges. In terms of mechanical properties, fibroin typically surpasses keratin in tensile strength and elasticity. Ker films are more prone to brittleness during storage and often require plasticizers or crosslinking to improve flexibility [37,38]. Another limitation is their water stability. SF films can degrade in aqueous environments unless treated to induce β-sheet structures, whereas Ker’s stability in water restricts its swelling properties, reducing its effectiveness in absorbing exudate [28,36]. The processing of these materials into consistent films can also be problematic. Fibroin solutions tend to gel over time, complicating scalability, while keratin requires precise control during extraction and casting to maintain its structural integrity [24,27]. Finally, the cost and scalability of producing these films, particularly fibroin, remain significant obstacles. The processes involved in extracting and purifying these proteins are labor-intensive and expensive, making large-scale production challenging [30,31].

## 4. Conclusions and Perspectives

In this review, we focused on natural protein films as potential platforms for wound healing and wound dressing applications. Specifically, we examined two proteins, keratin and fibroin, extracted from textile wastes. Both fibroin and keratin offer exceptional properties, including mechanical strength, biodegradability, biocompatibility, cell adhesion capabilities, and adaptability for processing into various formats, such as sponges, electrospun nanofibers, nanoparticles, and films. Furthermore, these natural proteins can be functionalized with a wide range of compounds with antimicrobial, antioxidant, and anti-inflammatory properties. We examined key fabrication techniques for SF and Ker films, such as solvent casting, spin coating, salt leaching, microfluidic spinning, and dip coating, and reviewed studies demonstrating their functionalization and application. The promising results to date support the potential of natural biopolymers in advancing wound healing systems and devices. Looking forward, keratin- and fibroin-based films hold significant promise for transforming wound care by mimicking extracellular matrix (ECM) properties. These materials enhance tissue regeneration through improved cell adhesion, proliferation, and controlled drug delivery. Modern fabrication methods, such as electrospinning and 3D printing, enable precise tailoring for specific wound types, while functionalization with bioactive agents further amplifies their therapeutic potential. Despite encouraging in vivo results—such as fibroin films loaded with insulin-like growth factor-1 (IGF-1) improving wound closure rates in diabetic models and keratin-based materials enhancing collagen synthesis—the transition to clinical trials remains a critical challenge. Regulatory barriers, scalability issues, and the limited predictive power of animal models for human wound environments impede clinical translation. Future research should prioritize standardized in vivo studies that replicate chronic wound conditions and consider comorbidities. Multidisciplinary collaborations between researchers, clinicians, and industry stakeholders will be essential to bridge the gap between laboratory findings and clinical applications. By addressing the current limitations in scaling and regulatory pathways, keratin- and fibroin-based films have the potential to revolutionize wound care, paving the way for more effective, sustainable, and personalized treatments.

## Figures and Tables

**Table 1:** Manufacturing methods for the preparation of films.

Methods	Advantages	Disadvantages
Solvent casting	Cost-effectiveness Feasibility Suitable mechanical properties Good homogeneity	Brittleness upon storage Difficult to scale up
Salt leaching	Easy and viable technique Tunable porosity and pore sizes	Not for water-soluble materials Not complete removal of salts
Spin coating	Thickness control via spin speed Thickness uniformity	Loss of material during spinning
Microfluidic spinning	Suitability for volatile compounds Reproducibility Capability of producing patterns	Costly technique Need to determine process parameters
Dip coating	Efficiency Ability to coat a wide variety of substrate materials	Risk of unbalanced coverage Susceptibility to turbulence

**Table 2:** Films with antimicrobial function.

	Mechanism of Action	Ref.
Antibiotics	Antimicrobial activity	[54]
Metal and metal oxide nanoparticles/inorganic nanomaterials	Avoid bacterial adhesion and biofilm formation through ROS generation	[55,56,57,58,59,60,61,62,63]
Honey	Sterilization Fosters endothelial growth	[64,65,66,67,68,69,70,71,72,73]
Chitosan	Antimicrobial and drug release agent	[8,74,75,76]
Phenolic compounds from plant extracts	Inhibit microbial proliferation	[77]
Antimicrobial peptides	Antibacterial Angiogenic	[78,79]
Photodynamic therapy	Antimicrobial through ROS generation	[80]

**Table 3:** Films with antioxidant function.

	Mechanism of Action	Ref.
Vitamin C (VC), epigallocatechin gallate (EGCG), and curcumin	Preservative Stabilizing	[84]
Curcumin	Preservative Stabilizing ROS scavenging Enhancing cellular proliferation and differentiation	[22,85,86,87,88,89,90,91]
Pomegranate extract	Drug release agent Reducing oxidative stress in cells	[92]
Chromophore	UV absorption	[93]
Keratoses	Iron-binding ability	[96]

**Table 4:** Growth factors.

	Mechanism of Action	Ref.
EGF	Increases keratinocyte proliferation and migration	[98]
IGF-1	Activation of the IGF1R pathway	[99,100,101]
Transgenic technology	Silkworm cocoons genetically engineered to produce human growth factors	[102]
Keratin-based products	Enhancing keratinocyte migration and collagen production	[103]

**Table 5:** Films with anti-inflammatory function.

	Mechanism of Action	Ref.
Modulation of films’ surface topography	Inducing differential macrophage polarization	[104]
Cytokines IFN-γ and IL-4	Modulating macrophage polarization	[105]
Neurotensin	Reducing inflammatory status Promoting fibroblast migration	[107,108]
ZnAl HTIc-DIK	COX-1 and COX-2 inhibition Antimicrobial activity	[109,110,111,112]
DIK	COX-1 and COX-2 inhibition Antimicrobial activity	[113]

## Data Availability

Not applicable.

## References

[1] Borbolla-Jiménez F.V., Peña-Corona S.I., Farah S.J., Jiménez-Valdés M.T., Pineda-Pérez E., Romero-Montero A., Del Prado-Audelo M.L., Bernal-Chávez S.A., Magaña J.J., Leyva-Gómez G. (2023). Films for Wound Healing Fabricated Using a Solvent Casting Technique. Pharmaceutics.

[2] Sheokand B., Vats M., Kumar A., Srivastava C.M., Bahadur I., Pathak S.R. (2023). Natural Polymers Used in the Dressing Materials for Wound Healing: Past, Present and Future. J. Polym. Sci..

[3] Çalamak S., Erdoǧdu C., Özalp M., Ulubayram K. (2014). Silk Fibroin Based Antibacterial Bionanotextiles as Wound Dressing Materials. Mater. Sci. Eng. C.

[4] Radulescu D.M., Andronescu E., Vasile O.R., Ficai A., Vasile B.S. (2024). Silk Fibroin-Based Scaffolds for Wound Healing Applications with Metal Oxide Nanoparticles. J. Drug Deliv. Sci. Technol..

[5] Pollini M., Paladini F. (2020). Bioinspired Materials for Wound Healing Application: The Potential of Silk Fibroin. Materials.

[6] Lujerdean C., Baci G.M., Cucu A.A., Dezmirean D.S. (2022). The Contribution of Silk Fibroin in Biomedical Engineering. Insects.

[7] Gupta B., Agarwal R., Alam M.S. (2010). Textile-Based Smart Wound Dressings. Indian J. Fibre Text. Res..

[8] Ganesan P. (2017). Natural and Bio Polymer Curative Films for Wound Dressing Medical Applications. Wound Med..

[9] El-Sayed H., Taleb M.A., Mowafi S. (2021). Potential Applications of Textile Wastes and By-Products in Preparation of Textile Auxiliaries. Egypt. J. Chem..

[10] Rockwood D.N., Preda R.C., Yücel T., Wang X., Lovett M.L., Kaplan D.L. (2011). Materials Fabrication from Bombyx Mori Silk Fibroin. Nat. Protoc..

[11] Numata K., Kaplan D.L. (2010). Silk-Based Delivery Systems of Bioactive Molecules. Adv. Drug Deliv. Rev..

[12] Sun W., Gregory D.A., Tomeh M.A., Zhao X. (2021). Silk Fibroin as a Functional Biomaterial for Tissue Engineering. J. Mol. Sci..

[13] Nguyen T.P., Nguyen Q.V., Nguyen V.H., Le T.H., Huynh V.Q.N., Vo D.V.N., Trinh Q.T., Kim S.Y., Van Le Q. (2019). Silk Fibroin-Based Biomaterials for Biomedical Applications: A Review. Polymers.

[14] Vidya M., Rajagopal S. (2021). Silk Fibroin: A Promising Tool for Wound Healing and Skin Regeneration. Int. J. Polym. Sci..

[15] Chilakamarry C.R., Mahmood S., Saffe S.N.B.M., Arifin M.A.B., Gupta A., Sikkandar M.Y., Begum S.S., Narasaiah B. (2021). Extraction and Application of Keratin from Natural Resources: A Review. 3 Biotech.

[16] Xu H., Cai S., Xu L., Yang Y. (2014). Water-Stable Three-Dimensional Ultrafine Fibrous Scaffolds from Keratin for Cartilage Tissue Engineering. Langmuir.

[17] Giannelli M., Barbalinardo M., Riminucci A., Belvedere K., Boccalon E., Sotgiu G., Corticelli F., Ruani G., Zamboni R., Aluigi A. (2021). Magnetic Keratin/Hydrotalcites Sponges as Potential Scaffolds for Tissue Regeneration. Appl. Clay Sci..

[18] Posati T., Ferroni C., Aluigi A., Guerrini A., Rossi F., Tatini F., Ratto F., Marras E., Gariboldi M.B., Sagnella A. (2018). Mild and Effective Polymerization of Dopamine on Keratin Films for Innovative Photoactivable and Biocompatible Coated Materials. Macromol. Mater. Eng..

[19] Aluigi A., Sotgiu G., Ferroni C., Duchi S., Lucarelli E., Martini C., Posati T., Guerrini A., Ballestri M., Corticelli F. (2016). Chlorin E6 Keratin Nanoparticles for Photodynamic Anticancer Therapy. RSC Adv..

[20] dos Santos F.V., Siqueira R.L., de Morais Ramos L., Yoshioka S.A., Branciforti M.C., Correa D.S. (2024). Silk Fibroin-Derived Electrospun Materials for Biomedical Applications: A Review. Int. J. Biol. Macromol..

[21] Savencu I., Iurian S., Porfire A., Bogdan C., Tomuță I. (2021). Review of Advances in Polymeric Wound Dressing Films. React. Funct. Polym..

[22] Alven S., Nqoro X., Aderibigbe B.A. (2020). Polymer-Based Materials Loaded with Curcumin for Wound Healing Applications. Polymers.

[23] Hodge J.G., Zamierowski D.S., Robinson J.L., Mellott A.J. (2022). Evaluating Polymeric Biomaterials to Improve next Generation Wound Dressing Design. Biomater. Res..

[24] Karki S., Kim H., Na S.J., Shin D., Jo K., Lee J. (2016). Thin Films as an Emerging Platform for Drug Delivery. Asian J. Pharm. Sci..

[25] Hou Q., Grijpma D.W., Feijen J. (2003). Porous Polymeric Structures for Tissue Engineering Prepared by a Coagulation, Compression Moulding and Salt Leaching Technique. Biomaterials.

[26] Draczynski Z., Kolesinska B., Latanska I., Sujka W. (2018). Preparation Method of Porous Dressing Materials Based on Butyric-Acetic Chitin Co-Polyesters. Materials.

[27] Aramwit P., Ratanavaraporn J., Ekgasit S., Tongsakul D., Bang N. (2015). A Green Salt-Leaching Technique to Produce Sericin/PVA/Glycerin Scaffolds with Distinguished Characteristics for Wound-Dressing Applications. J. Biomed. Mater. Res. B Appl. Biomater..

[28] Liu J., Huang R., Li G., Kaplan D.L., Zheng Z., Wang X. (2021). Generation of Nano-Pores in Silk Fibroin Films Using Silk Nanoparticles for Full-Thickness Wound Healing. Biomacromolecules.

[29] Sahu N., Panigrahi S. (2009). Fundamental Understanding and Modeling of Spin Coating Process: A Review. Indian J. Phys..

[30] Wasapinyokul K., Kaewpirom S., Boonsang S., Chuwongin S. Highly-Transparent Multi-Layered Spin-Coated Silk Fibroin Film. Proceedings of the AOPC 2017: Optoelectronics and Micro/Nano-Optics.

[31] Cheng J., Jun Y., Qin J., Lee S.H. (2017). Electrospinning versus Microfluidic Spinning of Functional Fibers for Biomedical Applications. Biomaterials.

[32] Brinker C.J. (2013). Dip Coating. Chemical Solution Deposition of Functional Oxide Thin Films.

[33] Fuest S., Smeets R., Gosau M., Aavani F., Knipfer C., Grust A.L.C., Kopp A., Becerikli M., Behr B., Matthies L. (2023). Layer-by-Layer Deposition of Regenerated Silk Fibroin—An Approach to the Surface Coating of Biomedical Implant Materials. ACS Biomater. Sci. Eng..

[34] Wang X., Hu X., Daley A., Rabotyagova O., Cebe P., Kaplan D.L. (2007). Nanolayer Biomaterial Coatings of Silk Fibroin for Controlled Release. J. Control. Release.

[35] Vepari C., Kaplan D.L. (2007). Silk as a Biomaterial. Prog. Polym. Sci..

[36] Yamauchi K., Yamauchi A., Kusunoki T., Kohda A., Konishi Y. (1996). Preparation of Stable Aqueous Solution of Keratins, and Physiochemical and Biodegradational Properties of Films. J. Biomed. Mater. Res..

[37] Tanabe T., Okitsu N., Yamauchi K. (2004). Fabrication and Characterization of Chemically Crosslinked Keratin Films. Mater. Sci. Eng. C.

[38] Lu Q., Hu X., Wang X., Kluge J.A., Lu S., Cebe P., Kaplan D.L. (2010). Water-Insoluble Silk Films with Silk I Structure. Acta Biomater..

[39] dos Santos F.V., Yoshioka S.A., Branciforti M.C. (2021). Large-Area Thin Films of Silk Fibroin Prepared by Two Methods with Formic Acid as Solvent and Glycerol as Plasticizer. J. Appl. Polym. Sci..

[40] Um I.C., Kweon H., Park Y.H., Hudson S. (2001). Structural Characteristics and Properties of the Regenerated Silk Fibroin Prepared from Formic Acid. Int. J. Biol. Macromol..

[41] Ma Y.-Y., Qi R.-R., Jia S.-Y., Wang Z.-H. Preparation and Characterization of Keratin-Cellulose Composite Films. Proceedings of the International Conference on Advanced Material Engineering.

[42] Vasconcelos A., Freddi G., Cavaco-Paulo A. (2008). Biodegradable Materials Based on Silk Fibroin and Keratin. Biomacromolecules.

[43] Um I.C., Kweon H.Y., Lee K.G., Park Y.H. (2003). The Role of Formic Acid in Solution Stability and Crystallization of Silk Protein Polymer. Int. J. Biol. Macromol..

[44] Rajkhowa R., Levin B., Redmond S.L., Li L.H., Wang L., Kanwar J.R., Atlas M.D., Wang X. (2011). Structure and Properties of Biomedical Films Prepared from Aqueous and Acidic Silk Fibroin Solutions. J. Biomed. Mater. Res. A.

[45] Sashina E.S., Novoselov N.P., Vnuchkin A.V., Golubikhin A.Y. (2007). Preparation and Properties of Films of Fibroin-Polyvinyl Alcohol Blends from Solutions in Hexafluoroisopropanol. Russ. J. Appl. Chem..

[46] Zhang X., Chen Z., Bao H., Liang J., Xu S., Cheng G., Zhu Y. (2019). Fabrication and Characterization of Silk Fibroin/Curcumin Sustained-Release Film. Materials.

[47] Sagnella A., Pistone A., Bonetti S., Donnadio A., Saracino E., Nocchetti M., Dionigi C., Ruani G., Muccini M., Posati T. (2016). Effect of Different Fabrication Methods on the Chemo-Physical Properties of Silk Fibroin Films and on Their Interaction with Neural Cells. RSC Adv..

[48] Brown J.E., Davidowski S.K., Xu D., Cebe P., Onofrei D., Holland G.P., Kaplan D.L. (2016). Thermal and Structural Properties of Silk Biomaterials Plasticized by Glycerol. Biomacromolecules.

[49] Belda Marín C., Egles C., Humblot V., Lalatonne Y., Motte L., Landoulsi J., Guénin E. (2021). Gold, Silver, and Iron Oxide Nanoparticle Incorporation into Silk Hydrogels for Biomedical Applications: Elaboration, Structure, and Properties. ACS Biomater. Sci. Eng..

[50] Ghalei S., Handa H. (2022). A Review on Antibacterial Silk Fibroin-Based Biomaterials: Current State and Prospects. Mater. Today Chem..

[51] Konop M., Rybka M., Drapała A. (2021). Keratin Biomaterials in Skin Wound Healing, an Old Player in Modern Medicine: A Mini Review. Pharmaceutics.

[52] Khajavi R., Rahimi M.K., Abbasipour M., Brendjchi A.H. (2016). Antibacterial Nanofibrous Scaffolds with Lowered Cytotoxicity Using Keratin Extracted from Quail Feathers. J. Bioact. Compat. Polym..

[53] Goyal S., Dotter M., Diestelhorst E., Storck J.L., Ehrmann A., Mahltig B. (2022). Extraction of Keratin from Wool and Its Use as Biopolymer in Film Formation and in Electrospinning for Composite Material Processing. J. Eng. Fibers Fabr..

[54] Yerra A., Mamatha D.M. (2021). Antibiotic-Based Silk Fibroin Films for Burn Wound Healing. Polym. Adv. Technol..

[55] Kushwaha A., Goswami L., Kim B.S. (2022). Nanomaterial-Based Therapy for Wound Healing. Nanomaterials.

[56] Dizaj S.M., Lotfipour F., Barzegar-Jalali M., Zarrintan M.H., Adibkia K. (2014). Antimicrobial Activity of the Metals and Metal Oxide Nanoparticles. Mater. Sci. Eng. C.

[57] Patil P.P., Meshram J.V., Bohara R.A., Nanaware S.G., Pawar S.H. (2018). ZnO Nanoparticle-Embedded Silk Fibroin-Polyvinyl Alcohol Composite Film: A Potential Dressing Material for Infected Wounds. New J. Chem..

[58] Patil P.P., Bohara R.A., Meshram J.V., Nanaware S.G., Pawar S.H. (2019). Hybrid Chitosan-ZnO Nanoparticles Coated with a Sonochemical Technique on Silk Fibroin-PVA Composite Film: A Synergistic Antibacterial Activity. Int. J. Biol. Macromol..

[59] Paladini F., Pollini M. (2019). Antimicrobial Silver Nanoparticles for Wound Healing Application: Progress and Future Trends. Materials.

[60] Cadinoiu A.N., Rata D.M., Daraba O.M., Ichim D.L., Popescu I., Solcan C., Solcan G. (2022). Silver Nanoparticles Biocomposite Films with Antimicrobial Activity: In Vitro and In Vivo Tests. Int. J. Mol. Sci..

[61] Panáček A., Kvítek L., Smékalová M., Večeřová R., Kolář M., Röderová M., Dyčka F., Šebela M., Prucek R., Tomanec O. (2018). Bacterial Resistance to Silver Nanoparticles and How to Overcome It. Nat. Nanotechnol..

[62] Patil S., Singh N. (2019). Antibacterial Silk Fibroin Scaffolds with Green Synthesized Silver Nanoparticles for Osteoblast Proliferation and Human Mesenchymal Stem Cell Differentiation. Colloids Surf. B Biointerfaces.

[63] Zhu G., Sun Z., Hui P., Chen W., Jiang X. (2021). Composite Film with Antibacterial Gold Nanoparticles and Silk Fibroin for Treating Multidrug-Resistant *E. coli*-Infected Wounds. ACS Biomater. Sci. Eng..

[64] Mandal M.D., Mandal S. (2011). Honey: Its Medicinal Property and Antibacterial Activity. Asian Pac. J. Trop. Biomed..

[65] Ranzato E., Martinotti S., Burlando B. (2012). Epithelial Mesenchymal Transition Traits in Honey-Driven Keratinocyte Wound Healing: Comparison among Different Honeys. Wound Repair Regen..

[66] Yang X., Fan L., Ma L., Wang Y., Lin S., Yu F., Pan X., Luo G., Zhang D., Wang H. (2017). Green Electrospun Manuka Honey/Silk Fibroin Fibrous Matrices as Potential Wound Dressing. Mater. Des..

[67] Scepankova H., Combarros-Fuertes P., Fresno J.M., Tornadijo M.E., Dias M.S., Pinto C.A., Saraiva J.A., Estevinho L.M. (2021). Role of Honey in Advanced Wound Care. Molecules.

[68] Almasaudi S. (2021). The Antibacterial Activities of Honey. Saudi J. Biol. Sci..

[69] Bizerra F.C., Da Silva P.I., Hayashi M.A.F. (2012). Exploring the Antibacterial Properties of Honey and Its Potential. Front. Microbiol..

[70] Minden-Birkenmaier B.A., Bowlin G.L. (2018). Honey-Based Templates in Wound Healing and Tissue Engineering. Bioengineering.

[71] Tashkandi H. (2021). Honey in Wound Healing: An Updated Review. Open Life Sci.

[72] Rajput M., Bhandaru N., Barui A., Chaudhary A., Paul R.R., Mukherjee R., Chatterjee J. (2014). Nano-Patterned Honey Incorporated Silk Fibroin Membranes for Improving Cellular Compatibility. RSC Adv..

[73] Nachiappan S. Silk Based Scaffolds in Combination with Honey and RhEGF for Diabetic Wound Healing. https://www.researchgate.net/publication/332670676_Silk_based_scaffolds_in_combination_with_honey_and_rhEGF_for_diabetic_wound_healing.

[74] Guang S., An Y., Ke F., Zhao D., Shen Y., Xu H. (2015). Chitosan/Silk Fibroin Composite Scaffolds for Wound Dressing. J. Appl. Polym. Sci..

[75] Rosewald M., Hou F.Y.S., m Mututuvari T., Harkins A., d Tran C. (2014). Cellulose-Chitosan-Keratin Composite Materials: Synthesis, Immunological and Antibacterial Properties. ECS Trans..

[76] Tran C.D., Mututuvari T.M. (2015). Cellulose, Chitosan, and Keratin Composite Materials. Controlled Drug Release. Langmuir.

[77] Basal G., Altıok D., Bayraktar O. (2010). Antibacterial properties of silk fibroin/chitosan blend films loaded with plant extract. Fibers Polym..

[78] Zhou W., Xie Z., Si R., Chen Z., Javeed A., Li J., Wu Y., Han B. (2023). Actinomycin-X2-Immobilized Silk Fibroin Film with Enhanced Antimicrobial and Wound Healing Activities. Int. J. Mol. Sci..

[79] Si R., Chen W., Chen J., Yang Y., Zhou W., Zhang Q., Chen C., Han B. (2023). Green Chemistry Fabrication of Durable Antimicrobial Peptide-Immobilized Silk Fibroin Films for Accelerated Full-Thickness Wound Healing. Mater. Today Chem..

[80] Aluigi A., Sotgiu G., Torreggiani A., Guerrini A., Orlandi V.T., Corticelli F., Varchi G. (2015). Methylene Blue Doped Films of Wool Keratin with Antimicrobial Photodynamic Activity. ACS Appl. Mater. Interfaces.

[81] Sanchez M.C., Lancel S., Boulanger E., Neviere R. (2018). Targeting Oxidative Stress and Mitochondrial Dysfunction in the Treatment of Impaired Wound Healing: A Systematic Review. Antioxidants.

[82] Dunnill C., Patton T., Brennan J., Barrett J., Dryden M., Cooke J., Leaper D., Georgopoulos N.T. (2017). Reactive Oxygen Species (ROS) and Wound Healing: The Functional Role of ROS and Emerging ROS-Modulating Technologies for Augmentation of the Healing Process. Int. Wound J..

[83] Fadilah N.I.M., Phang S.J., Kamaruzaman N., Salleh A., Zawani M., Sanyal A., Maarof M., Fauzi M.B. (2023). Antioxidant Biomaterials in Cutaneous Wound Healing and Tissue Regeneration: A Critical Review. Antioxidants.

[84] Luo T., Yang L., Wu J., Zheng Z., Li G., Wang X., Kaplan D.L. (2016). Stabilization of Natural Antioxidants by Silk Biomaterials. ACS Appl. Mater. Interfaces.

[85] Mohanty C., Sahoo S.K. (2017). Curcumin and Its Topical Formulations for Wound Healing Applications. Drug Discov. Today.

[86] Liang G., Yang S., Zhou H., Shao L., Huang K., Xiao J., Huang Z., Li X. (2009). Synthesis, Crystal Structure and Anti-Inflammatory Properties of Curcumin Analogues. Eur. J. Med. Chem..

[87] Ak T., Gülçin I. (2008). Antioxidant and Radical Scavenging Properties of Curcumin. Chem. Biol. Interact..

[88] Mun S.H., Joung D.K., Kim Y.S., Kang O.H., Kim S.B., Seo Y.S., Kim Y.C., Lee D.S., Shin D.W., Kweon K.T. (2013). Synergistic Antibacterial Effect of Curcumin against Methicillin-Resistant Staphylococcus Aureus. Phytomedicine.

[89] Joe B., Vijaykumar M., Lokesh B.R. (2004). Biological Properties of Curcumin-Cellular and Molecular Mechanisms of Action. Crit. Rev. Food Sci. Nutr..

[90] Akbik D., Ghadiri M., Chrzanowski W., Rohanizadeh R. (2014). Curcumin as a Wound Healing Agent. Life Sci..

[91] Li C., Luo T., Zheng Z., Murphy A.R., Wang X., Kaplan D.L. (2015). Curcumin-Functionalized Silk Materials for Enhancing Adipogenic Differentiation of Bone Marrow-Derived Human Mesenchymal Stem Cells. Acta Biomater..

[92] Barbalinardo M., Giannelli M., Forcini L., Luppi B., Donnadio A., Navacchia M.L., Ruani G., Sotgiu G., Aluigi A., Zamboni R. (2022). Eco-Sustainable Silk Fibroin/Pomegranate Peel Extract Film as an Innovative Green Material for Skin Repair. Int. J. Mol. Sci..

[93] Chen B., Xing Y., Yu W., Liu H. (2018). Wool Keratin and Silk Sericin Composite Films Reinforced by Molecular Network Reconstruction. J. Mater. Sci..

[94] Wright J.A., Richards T., Srai S.K.S. (2014). The Role of Iron in the Skin and Cutaneous Wound Healing. Front. Pharmacol..

[95] Aroun A., Zhong J.L., Tyrrell R.M., Pourzand C. (2012). Iron, Oxidative Stress and the Example of Solar Ultraviolet A Radiation. Photochem. Photobiol. Sci..

[96] Anceschi A., Patrucco A., Bhavsar P., Zoccola M., Tessari M., Erbazzi L., Zamboni P. (2023). Keratose Self-Cross-Linked Wound Dressing for Iron Sequestration in Chronic Wounds. ACS Omega.

[97] Zhang Y., Atala A. (2018). Regenerative Medicine of the Bladder. Principles of Regenerative Medicine.

[98] Gil E.S., Panilaitis B., Bellas E., Kaplan D.L. (2013). Functionalized Silk Biomaterials for Wound Healing. Adv. Healthc. Mater..

[99] Lin M.J., Lu M.C., Chang H.Y. (2021). Sustained Release of Insulin-like Growth Factor-1 from Bombyx Mori L. Silk Fibroin Delivery for Diabetic Wound Therapy. Int. J. Mol. Sci..

[100] Garoufalia Z., Papadopetraki A., Karatza E., Vardakostas D., Philippou A., Kouraklis G., Mantas D. (2021). Insulin-like Growth Factor-I and Wound Healing, a Potential Answer to Non-Healing Wounds: A Systematic Review of the Literature and Future Perspectives. Biomed. Rep..

[101] Lin M.J., Lu M.C., Chan Y.C., Huang Y.F., Chang H.Y. (2021). An Insulin-like Growth Factor-1 Conjugated Bombyx Mori Silk Fibroin Film for Diabetic wound Healing: Fabrication, Physicochemical Property Characterization, and Dosage Optimization in Vitro and in Vivo. Pharmaceutics.

[102] Wang S.L., Li X.W., Xu W., Yu Q.Y., Fang S.M. (2024). Advances of Regenerated and Functionalized Silk Biomaterials and Application in Skin Wound Healing. Int. J. Biol. Macromol..

[103] Tang L., Sierra J.O., Kelly R., Kirsner R.S., Li J. (2012). Wool-Derived Keratin Stimulates Human Keratinocyte Migration and Types IV and VII Collagen Expression. Exp. Dermatol..

[104] Hu D., Li T., Bian H., Liu H., Wang P., Wang Y., Sun J. (2024). Silk Films with Distinct Surface Topography Modulate Plasma Membrane Curvature to Polarize Macrophages. Mater. Today Bio.

[105] Reeves A.R.D., Spiller K.L., Freytes D.O., Vunjak-Novakovic G., Kaplan D.L. (2015). Controlled Release of Cytokines Using Silk-Biomaterials for Macrophage Polarization. Biomaterials.

[106] Moura L.I.F., Dias A.M.A., Suesca E., Casadiegos S., Leal E.C., Fontanilla M.R., Carvalho L., de Sousa H.C., Carvalho E. (2014). Neurotensin-Loaded Collagen Dressings Reduce Inflammation and Improve Wound Healing in Diabetic Mice. Biochim. Biophys. Acta Mol. Basis Dis..

[107] Da Silva L.P., Neves B.M., Moura L., Cruz M.T., Carvalho E. (2014). Neurotensin Decreases the Proinflammatory Status of Human Skin Fibroblasts and Increases Epidermal Growth Factor Expression. Int. J. Inflam..

[108] Lemos C.N., Cubayachi C., Dias K., Mendonça J.N., Lopes N.P., Furtado N.A.J.C., Lopez R.F.V. (2018). Iontophoresis-Stimulated Silk Fibroin Films as a Peptide Delivery System for Wound Healing. Eur. J. Pharm. Biopharm..

[109] Posati T., Giuri D., Nocchetti M., Sagnella A., Gariboldi M., Ferroni C., Sotgiu G., Varchi G., Zamboni R., Aluigi A. (2018). Keratin-Hydrotalcites Hybrid Films for Drug Delivery Applications. Eur. Polym. J..

[110] Goh C.F., Lane M.E. (2014). Formulation of Diclofenac for Dermal Delivery. Int. J. Pharm..

[111] Salem-Milani A., Balaei-Gajan E., Rahimi S., Moosavi Z., Abdollahi A., Zakeri-Milani P., Bolourian M., Salem Milani A. (2013). Antibacterial Effect of Diclofenac Sodium on Enterococcus Faecalis. J. Dent..

[112] Dutta N.K., Dastidar S.G., Kumar A., Mazumdar K., Ray R., Chakrabarty A.N. (2004). Antimycobacterial activity of the antiinflammatory agent diclofenac sodium, and its synergism with streptomycin. Braz. J. Microbiol..

[113] Cui L., Gong J., Fan X., Wang P., Wang Q., Qiu Y. (2013). Transglutaminase-Modified Wool Keratin Film and Its Potential Application in Tissue Engineering. Eng. Life Sci..

